# Changes in the vibration sensitivity and pressure pain thresholds in patients with burning mouth syndrome

**DOI:** 10.1371/journal.pone.0197834

**Published:** 2018-05-21

**Authors:** Brenda de Souza Moura, Natália dos Reis Ferreira, Marcos F. DosSantos, Maria Elisa Rangel Janini

**Affiliations:** 1 Departamento de Patologia e Diagnóstico Oral, Faculdade de Odontologia, Universidade Federal do Rio de Janeiro, Rio de Janeiro, Brazil; 2 Programa de Pós-Graduação em Radiologia, Faculdade de Medicina, Universidade Federal do Rio de Janeiro, Rio de Janeiro, Brazil; 3 Laboratório de Morfogênese Celular (LMC), Instituto de Ciências Biomédicas (ICB), Universidade Federal do Rio de Janeiro, Rio de Janeiro, Brazil; Weill Cornell Medicine-Qatar, QATAR

## Abstract

**Objective:**

To investigate the presence of changes in vibration detection and pressure pain threshold in patients with burning-mouth syndrome (BMS).

**Design of the study:**

Case-control study. The sample was composed of 30 volunteers, 15 with BMS and 15 in the control group. The pressure-pain threshold (PPT) and vibration-detection threshold (VDT) were examined. The clinical evaluation was complemented with the McGill Pain Questionnaire (MPQ), Douleur Neuropathique 4 (DN4) and Beck Depression and Anxiety Inventories (BDI and BAI, respectively).

**Results:**

BMS subjects showed a statistically significant higher PPT in the tongue (p = 0.002), right (p = 0.001) and left (p = 0.004) face, and a significant reduction of the VDT in the tongue (p = 0.013) and right face (p = 0.030). Significant differences were also found when comparing the PPT and the VDT of distinct anatomical areas. However, a significant interaction (group × location) was only for the PPT. BMS subjects also showed significantly higher levels of depression (p = 0.01), as measured by the BDI, compared to controls; and a significant inverse correlation between the VDT in the left face and anxiety levels was detected.

**Conclusions:**

The study of somatosensory changes in BMS and its correlations with the clinical features as well as the levels of anxiety and depression expands current understanding of the neuropathic origin and the possible contribution of psychogenic factors related to this disease.

## Introduction

Burning-mouth syndrome (BMS) is usually described as a burning sensation in the oral and perioral regions, in the absence of clinical and laboratory findings [1]. The International Association for the Study of Pain (IASP) has defined BMS as an “intraoral chronic burning pain wherein local or systemic causes cannot be identified.” In most patients, the burning-mouth sensation affects more than one anatomical location, usually with bilateral representation [2]. BMS affects mostly post-menopausal women and occurs mainly in the tongue. Other regions may also be affected [3–6].

Several factors may be involved in the BMS etiology. Among them are local and systemic causes, including endocrine, immunological and nutritional changes, as well as psychogenic factors [7,8,9]. Anxiety, depression and “cancerophobia” are the psychological features most frequently reported in the literature [2,8,10,11]; and in most cases, precede the appearance of BMS symptoms [11]. Nonetheless, the etiology and pathophysiology of BMS are still unclear and constitute an enormous challenge for health-care providers and researchers [1, 12].

Current literature points toward a neuropathic origin for BMS, involving both the peripheral and central nervous systems [12–16]. For example, neuropathy of small trigeminal fibers, characterized by a significant loss of epithelium and sub-papillary tongue nerve fibers, has been observed in BMS patients [17]. Therefore, the study of the clinical characteristics as well as the sensory changes associated with BMS is crucial to the understanding of its pathophysiology.

Many questionnaires have been developed and validated to investigate the different clinical aspects of chronic pain. Among them are the McGill Pain Questionnaire (MPQ) and the DN4 (Douleur Neuropathique en 4 questions) neuropathic pain questionnaire, which have also been applied in clinical evaluation of BMS patients [18–20]. Other inventories or self-assessment scales have been applied to specifically examine the presence of anxiety and depression disorders in chronic-pain patients, such as Hamilton’s Anxiety (HAM-A) and Depression (HDM-D) rating scales and Beck’s Anxiety (BAI) and Depression (BDI) Inventories [21].

Besides the use of questionnaires, the presence of sensory changes related to chronic pain can be assessed in a standardized manner through a quantitative sensory test (QST) [14,15,22]. QST is an important tool for evaluation of the somatosensory function in patients with chronic neuropathic and nociceptive pain. This test can be defined as the analysis of the detection (perception) and/or pain thresholds, in response to different categories of external stimuli applied directly to the skin or mucosa, in an ascending and/or descending order of magnitude [23].

The complete QST protocol assesses the function of the Aδ, Aβ and C nerve fibers, through physical stimulation. This method is an important tool to establish normative values of sensitivity to different types of peripheral stimuli [24]. The protocol also provides parameters for evaluating the sensory changes (hyperalgesia, allodynia and dysesthesia) often found in neuropathic-pain patients [25]. This method has been applied recently in the study of BMS [14, 15, 22]. An adapted protocol has been developed for intraoral investigation [25]. Nevertheless, little is known regarding the presence and extent of changes in the somatosensory processing of BMS sufferers. For instance, variations in the vibration sensitivity as well as the pressure-pain threshold have not yet been studied in BMS patients.

The goals of this study were: first–to assess the presence of changes in the vibration-detection threshold (VDT) and pressure-pain threshold (PPT) in patients with BMS, when compared to healthy subjects; and second–to correlate the fluctuations in the VDT and PPT with the clinical data, represented by the chief complaint of BMS, together with the levels of associated anxiety and depression.

## Material and methods

### Research volunteers

This was a single-center, unblinded, case-control study. Thirty volunteers were recruited for this study, 15 BMS patients and 15 healthy volunteers (control group), matched for age and gender.

The diagnosis of BMS participants was carried out based on a meticulous clinical investigation of the oral cavity in order to exclude local causes that could be related to the clinical complaint, combined with a laboratory analysis to eliminate any systemic cause of burning or sore mouth. The symptoms of all BMS patients met the criteria listed in the International Classification of Headache Disorders (ICHD-3), third edition [26].

Inclusion criteria were: diagnosis of BMS lasting for at least six months and age over 18 years. Exclusion criteria were: the presence of any injury to the oral mucosa, patients younger than 18 years, cognitive deficit, systemic disorders, chronic pain syndromes, continuous use of medication that may alter or compromise the somatosensory function (e.g. anticonvulsants or antiepileptics) and pregnancy.

This study was carried out in accordance with the bioethical rules for studies involving human beings of the WMA (World Medical Association)—Declaration of Helsinki (1964) and later versions and National Institutes of Health, United States. The protocol was approved by the Ethics Committee of the Clementino Fraga Filho Hospital of the Federal University of Rio de Janeiro. All BMS patients were recruited from the Department of Oral Pathology and Diagnosis, School of Dentistry, Federal University of Rio de Janeiro. Healthy volunteers were recruited through advertisements at the Federal University of Rio de Janeiro and local community. All volunteers were previously informed regarding the nature of the study as well as its inclusion and exclusion criteria, and signed a written informed consent form.

The research volunteers were divided into two groups: BMS and control group. All tests and questionnaires for each patient were carried out on the same day. The total duration of the battery of tests and questionnaires was around one hour for each volunteer.

### Questionnaires

First, all BMS patients described the intensity of the burning-mouth sensation, through a Visual Analogue Scale (VAS). Following this step, each subject answered four specific standardized questionnaires, all validated for use in Portuguese, i.e., the MPQ, DN4, BAI and BDI [19, 27, 28]. The Portuguese versions of the MPQ and DN4 are available as supplemental materials (S1 and S2 Figs). MPQ and DN4 were only applied to BMS patients.

The MPQ questionnaire was developed to assess the dimensions of pain, through its descriptors, divided into 20 categories. The descriptors are divided as follows: sensory (S), affective (A) and evaluative (E) dimensions, as well as descriptors that do not fall into any of these categories and are characterized as miscellaneous (M). The sum of values obtained in each category provides the pain rating index (PRI, T). The MPQ also allows researchers to obtain qualitative information through the present pain intensity (PPI), on a scale from 0 to 5 (0-no pain, 1-mild, 2-discomforting, 3-distressing, 4-horrible and 5-excruciating) [20,29].

Regarding the BAI and BDI questionnaires, in addition to the total score (minimum of 0 and maximum of 63 for each test), each subject can be classified in one of four levels of anxiety or depression: minimum, mild, moderate or severe [21, 27].

DN4 is a screening tool for neuropathic-pain patients. This questionnaire is divided into four parts: the first part contains three items that match the description of the pain; the second part contains four items, all related to the presence of paresthesia/dysesthesia in the painful area; the third part has four items related to sensory deficits; and the fourth part consists of an item related to evoked pain [18, 19].

### QST

This study followed the protocol developed by the German Network on Neuropathic Pain (DFNS) for QST [25].

The measurements were made on both sides of the face, in the area of the mandibular division of the trigeminal nerve (V3), on the gingival mucosa of the upper premolar region, and on the apex of the tongue [24]. The specific objective of the QST was to detect variations in the vibration and pressure sensitivity related to BMS.

### Vibration detection threshold (VDT)

VDT evaluates the function of Aß fibers. Here, the VDT was assessed with a Rydel-Seiffer graduated tuning fork (64 Hz, 8/8 scale). During each test, the fork was set in motion and placed over an osseous anatomical structure (zygomatic arch or the gingival mucosa of the upper premolar region) or over the apex of the tongue. Each subject was instructed to inform when the vibration was no longer detectable. The values were obtained using a 9-point scale (0–8) with an accuracy of 1/2 unit. The arithmetic mean of three consecutive records was then calculated, thus establishing the VDT for each anatomical region of each subject [24].

### Pressure pain threshold (PPT)

PPT was used to test the deep pain sensitivity, which is mediated by either C or Aδ fibers. To evaluate the PPT, a digital pressure algometer (model FPX 25, Wagner Instruments, USA), with two tip sizes, was used. The tip with a surface area of 1 cm^2^ was used to measure the PPT in the masseter muscle and apex of the tongue, while the tip with a surface area of 0.18 cm^2^ (Ø 4.8 mm) was used to obtain the PPT in the gingival mucosa of the upper premolar region. During each test, the pressure was increased gradually at a rate of 50 kPa/s. Each subject was instructed to manually inform when a discomforting sensation started to appear, so that the examiner could interrupt the exam and record the value obtained. The PPT was determined as the arithmetic mean of the three records [24].

### Statistical analysis

Given the exploratory nature of this study, a convenience sample comprising 15 subjects with BMS and 15 healthy subjects was used. Initially, a descriptive evaluation of the variables was performed. The data were expressed as mean and standard deviation (SD). To accomplish the first objective, an inferential statistical analysis, consisting of the investigation of possible differences in the QST (PPT and VDT) variables between BMS subjects and healthy volunteers, was conducted in the five regions of the head examined (apex of the tongue, bilateral gingival mucosa of the upper premolar region, and bilateral face). To conclude the second objective, a correlation between clinical variables obtained with the questionnaires (DN4, McGill, BAI and BDI) and with the VAS and QST data, was calculated only for individuals with BMS. Mean age was compared between the two groups by Student's two-tailed t test for two independent samples. All statistical analyses were performed using Stata software, version 14 (StataCorp, College Station, TX, USA).

To ensure a normal distribution of the data, a logarithmic transformation (Log10) was applied to all QST parameters. The significance level was set at 5%. Fisher’s exact test was used to compare the ordinal qualitative variables (BAI score and BDI score) between the two groups. For comparisons between the continuous quantitative QST variables, a factorial (two-way) repeated-measures ANOVA was applied, considering the group (patient or control) as the between-subjects factor and the area tested (tongue, bilateral gingival mucosa of the upper premolar region or bilateral face) as the within-subjects factor. The normal distribution of the residuals was investigated using the Shapiro-Wilk test (p > 0.05). Sphericity was tested by applying the Huynh-Feldt (H-F) correction when the estimated sphericity, epsilon (ε), of Greenhouse-Geisser (G-G) was higher than 0.75, while the most conservative GG correction was applied when ε < 0.75 [30]. Homoscedasticity was investigated by the Levene test. In the presence of significant interaction between the factors, simple main-effects analyses were conducted, to separate the interaction (group × area) found. Contrasts of marginal linear predictions allowed comparison between the marginal means of each group.

To establish the degree of difference between BMS patients and healthy subjects, a Z-score (standard score) was calculated using the formula: Z-score = (Value patient–Value control) / SD control) [15]. This score provided a somatosensory profile of BMS patients, regardless of the units of the different QST measures (e.g. VDT and PPT). A Z-score < –1.96 or > 1.96 was interpreted as a significant change in the somatosensory function. Data obtained from BMS patients was compared to the data obtained from the control group but not to reference values. Spearman’s rank correlation coefficient was used to estimate the degree of association between the clinical measures of pain and the QST data in BMS patients.

## Results

The results of the descriptive evaluation are shown in Tables 1–4. Fifteen patients with BMS and 15 healthy subjects, paired for age and gender, were studied. Each group was composed of 12 women (80% of the sample) and 3 men (20% of the sample). The mean age in the BMS group was 64.4 and in the control group, 61.3 years. As anticipated, there was no statistically significant difference in the mean age of the groups (t = 0.9573, p = 0.3466).

**Table 1:** Quantitative variables in the group of BMS patients and control group; N = number of participants, S.D. = standard deviation. Comparison refers to Student's two-tailed t test for two independent samples (age), Fisher’s exact test (BAI and BDI) or simple main effect analysis (QST variables). To = tongue, Fa = face, Gin = gingival mucosa of the upper premolar region, R = right side, L = left side.

		BMS			Controls		Comparison
Variable	N	Mean	S.D.	N	Mean	S.D.	p value
**Age**	15	64.4	8.8	15	61.3	8.7	0.3466
**BAI scores**	12	13.8	9.2	15	7.5	6.7	0.084
**BDI scores**	12	12.2	9.3	15	4.3	4.3	0.01
**PPT-To**	12	0.799	0.316	15	0.450	0.169	0.002
**PPT-Gin-R**	12	0.462	0.244	15	0.493	0.225	0.624
**PPT-Gin-L**	12	0.488	0.289	15	0.368	0.145	0.340
**PPT-Fa-R**	13	1.254	0.459	15	0.741	0.376	0.001
**PPT-Fa-L**	13	1.177	0.370	15	0.744	0.369	0.004
**VDT-To**	13	5.8	2.0	15	7.2	1.2	0.013
**VDT-Gin-R**	13	6.2	1.5	15	7.4	1.0	0.063
**VDT-Gin-L**	13	6.7	1.3	15	7.4	0.8	0.279
**VDT-Fa-R**	15	5.1	1.9	15	6.0	1.4	0.030
**VDT-Fa-L**	15	5.4	1.4	15	5.7	1.7	0.584

**Table 2:** Pain variables in the group of BMS patients; N = number of participants, S.D. = standard deviation, Min. = minimum values, Max. = maximum values.

Variable	N	Mean	S.D.	Min.	Max.
**DN4**	15	61.3	8.7	1	6
**VAS**	15	7.5	6.7	0.1	10
**McGill (S)**	15	4.3	4.3	6	29
**McGill (A)**	15	0.450	0.169	0	10
**McGill (E)**	15	0.493	0.225	0	5
**McGill M (A)**	15	0.368	0.145	0	7
**McGill (A/E)**	15	0.741	0.376	0	5
**McGill M (T)**	15	0.744	0.369	0	11
**McGill PRI (T)**	15	7.2	1.2	9	51
**DN4**	15	7.4	1.0	1	6
**VAS**	15	7.4	0.8	0.1	10
**McGill (S)**	15	6.0	1.4	6	29
**McGill (A)**	15	5.7	1.7	0	10

**Table 3:** BAI levels in the BMS patients (above) and control group (below).

Group	BAI	Frequency	Percentage (%)
**BMS**	Minimum	6	50
**BMS**	Mild	2	16.67
**BMS**	Moderate	4	33.33
**Total**		12	
**Controls**	Minimal	13	86.67
**Controls**	Moderate	2	13.33
**Total**		15	

**Table 4:** BDI in the BMS patients (above) and control group (below).

Group	BDI	Frequency	Percentage (%)
**BMS**	Minimum	7	58.33
**BMS**	Mild	2	16.67
**BMS**	Moderate	2	16.67
**BMS**	Severe	1	8.33
**Total**		12	
**Controls**	Minimum	15	100
**Total**		15	

All BMS patients reported a burning sensation on the tongue. Eight of them (53%) reported burning only at this location. The other seven patients (47%) reported that the burning sensation occurred concomitantly in other parts of the oral cavity, including the gingiva, palate and lips. The clinical exam of the oral cavity did not reveal any sign of lesion or oral disease in both groups.

The BMS patients and controls showed no statistically significant difference in levels of anxiety as measured by the BAI. However, the group of patients with BMS showed a significantly higher degree of depression as measured by the BDI, compared to the control group (p = 0.01). Approximately 20% of patients with BMS had levels suggestive of moderate or severe depression. On the other hand, in the control group, the BDI classification was always minimal.

**Fig 1:**
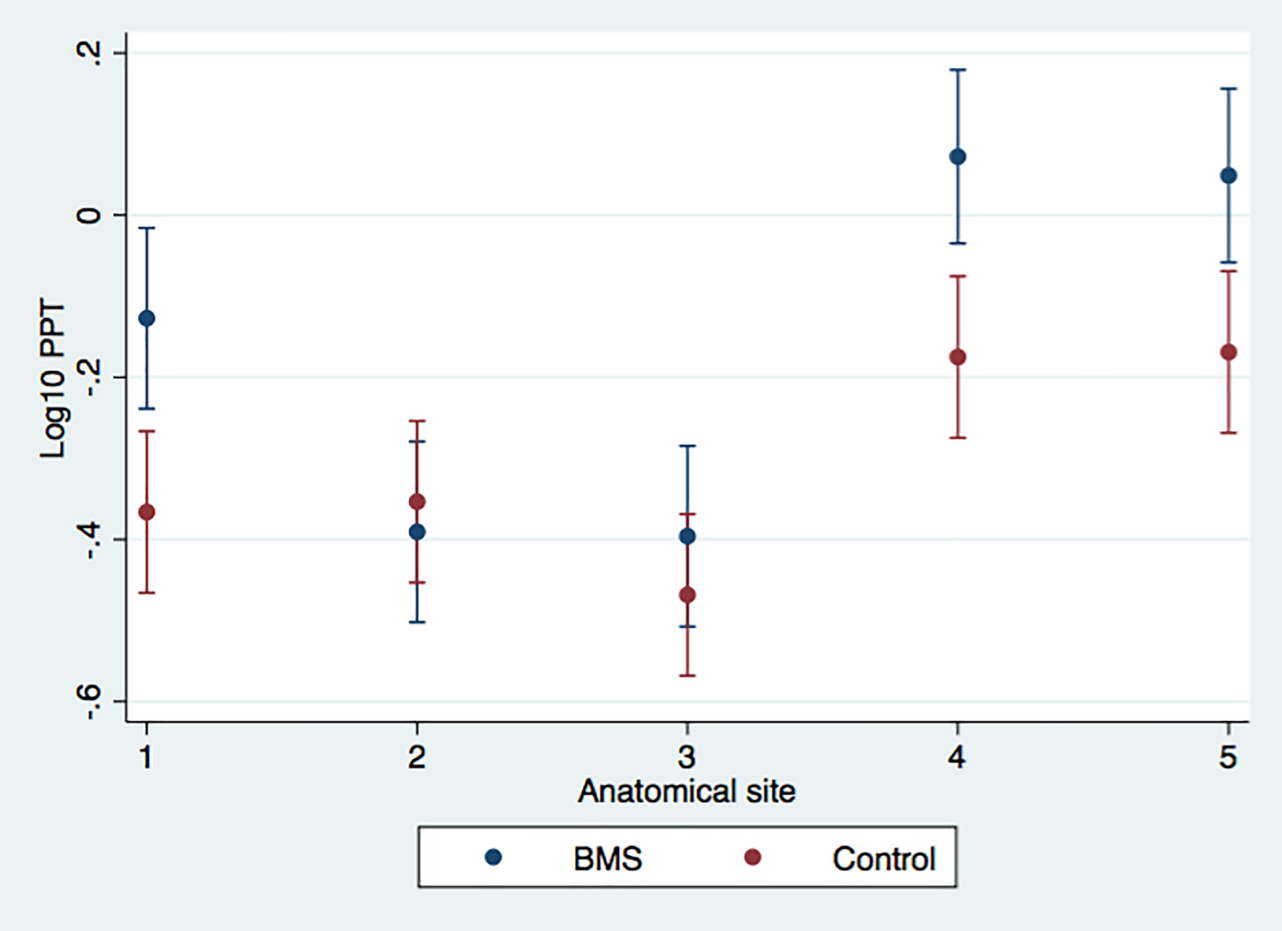
Adjusted predictions considering PPT measures (log PPT) in each anatomical area (1 = tongue, 2 = right gingival mucosa of upper premolar region, 3 = left gingival mucosa of upper premolar region, 4 = right side of face, and 5 = left side of face) in the two groups. Bars represent 95% confidence intervals.

**Fig 2:**
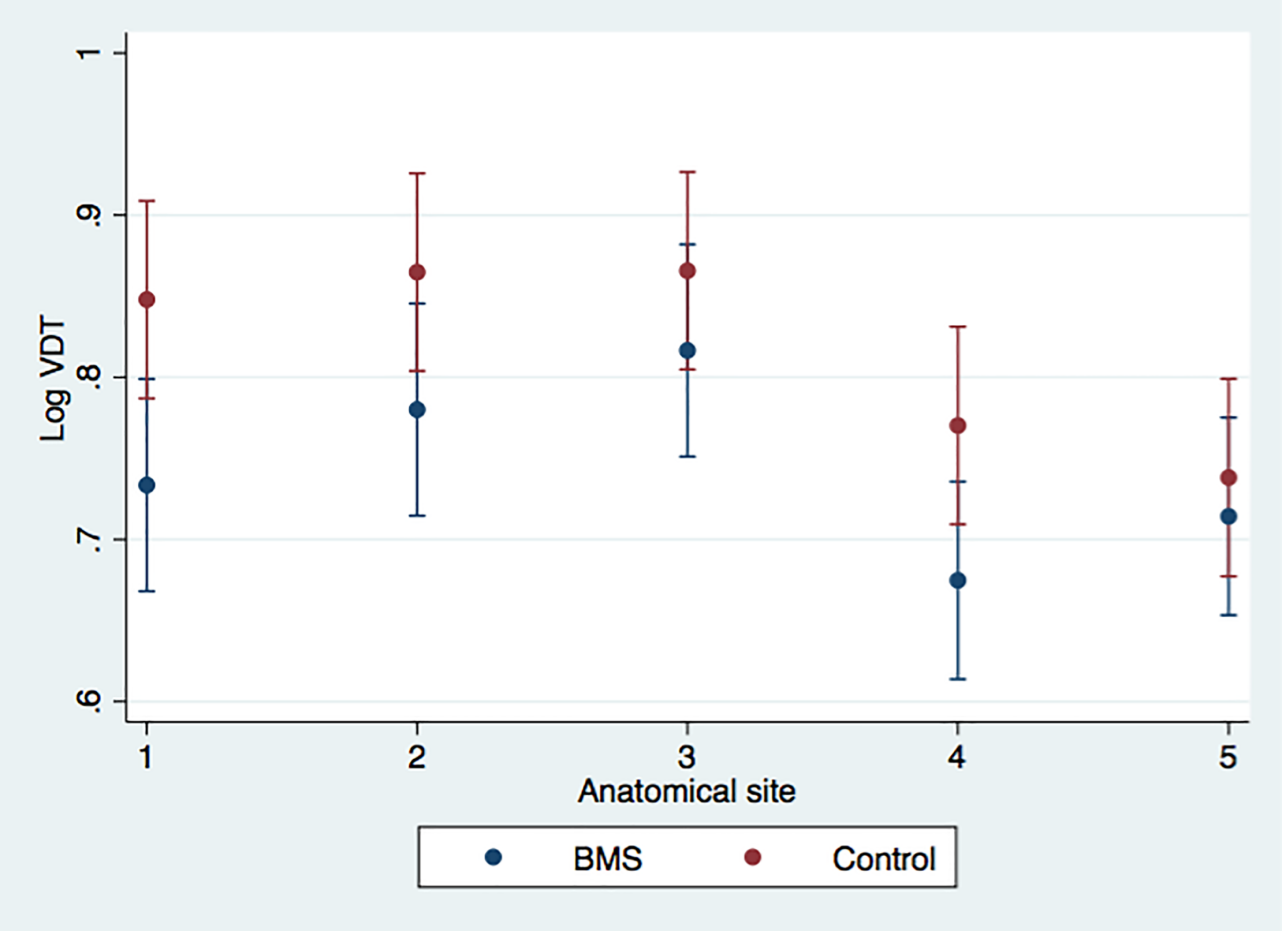
Adjusted predictions considering VDT measures (log PPT) in each anatomical area (1 = tongue, 2 = right gingival mucosa of upper premolar region, 3 = left gingival mucosa of upper premolar region, 4 = right side of face, and 5 = left side of face) in the two groups. Group 1 = BMS patients, group 2 = control group. Bars represent 95% confidence intervals.

A significant interaction (group × location) was found for the PPT, F (4.101) = 5.31, p = 0.0034, GG correction; but not for the VDT F (4.106) = 1.34, p = 0.2680, GG correction. Simple main effects analyses revealed a significant increase in the PPT of the tongue (p = 0.002), right side of the face (p = 0.001), and left side of the face (p = 0.004) in BMS patients. Conversely, the VDT was significantly lower on the tongue (p = 0.013) and on the right side of the face (p = 0.030) in subjects with BMS. These results are illustrated in Figs 1 and 2.

Comparing the PPT measures in the different areas analyzed revealed significant differences in nearly all anatomical regions of the two groups, at a 1% significance level in patients and a 5% level in the control group. Exceptions were found in the comparisons of PPTs between the right and left sides of the face in BMS patients (p = 0.675) and in the control subjects (p = 0.907), between the right and left sides of the gingival mucosa in BMS patients (p = 0.926), and between the tongue PPT and the right side of the gingival mucosa in the control subjects (p = 0.805).

VDT values also differed between several areas in the two groups, at a 5% significance level, except for the following comparisons: left and right sides of the face in BMS patients (p = 0.186) and in control subjects (p = 0.281), left and right sides of the gingival mucosa in patients (p = 0.255) and in controls (p = 0.977), tongue and left side of the face (p = 0.335) and tongue and right side of the gingival mucosa in patients (p = 0.146), as well as between the tongue VDT and the VDT of the right (p = 0569) and left sides of the gingiva (p = 0.549) in controls.

The Z-scores corroborate the results of the comparison between the two groups described above. In the evaluation of tongue sensitivity, 62% of subjects with BMS exhibited a significant functional increase in the PPT, while 31% of subjects showed a significant reduction in vibration sensitivity as measured by the VDT. The PPT function was also significantly higher on the right side of the face in 23% of individuals with BMS, while a significantly lower VDT function was observed in the same anatomical area in 33% of patients with BMS. The Z-score results for PPT and VDT, in each region, are shown in S3 Fig.

**Fig 3:**
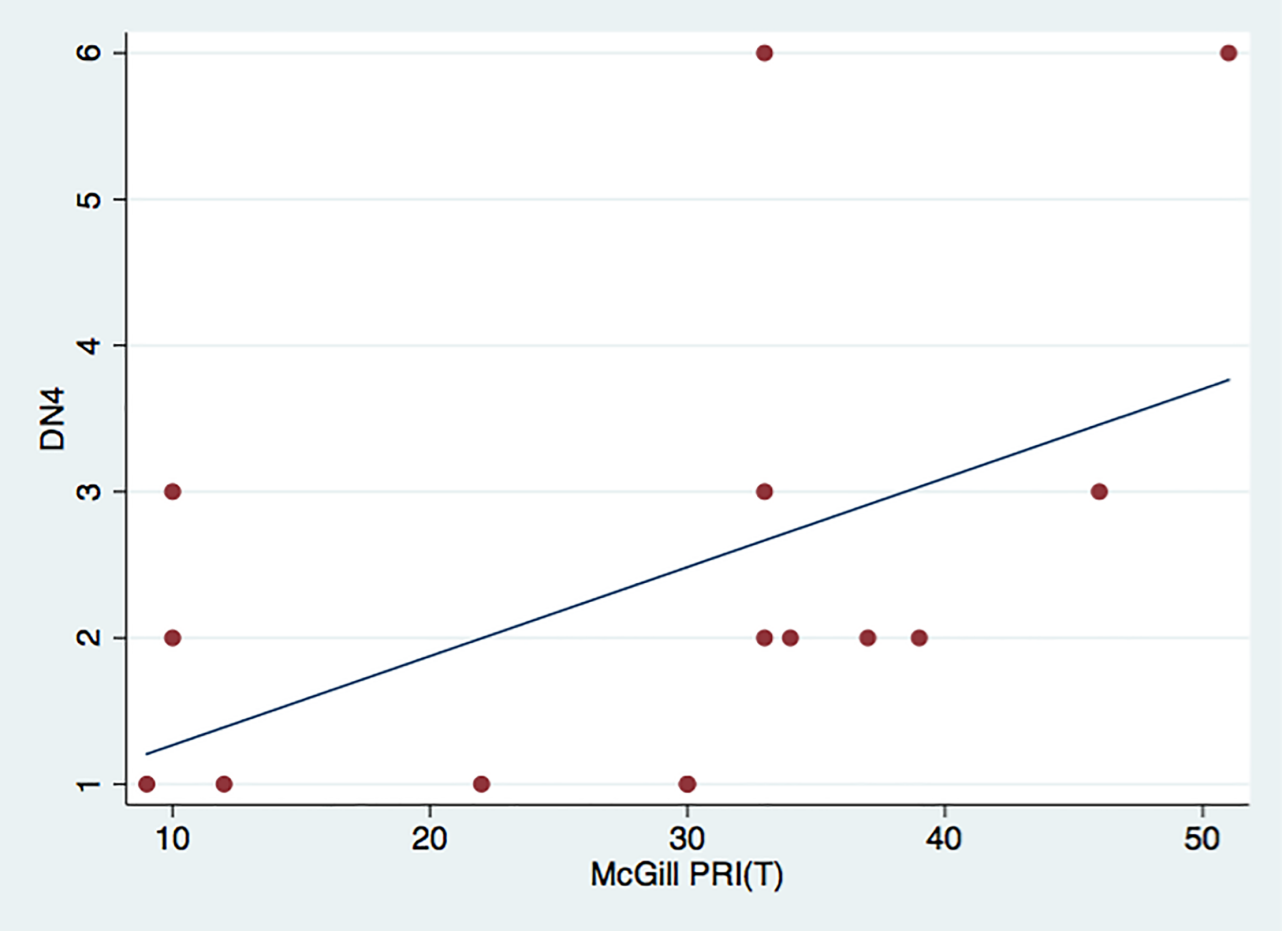
Scatter plot displaying direct correlation between McGill PRI (T) and DN4 scores. For visualization purposes, the line illustrates the trend of the relationship between the two variables.

**Fig 4:**
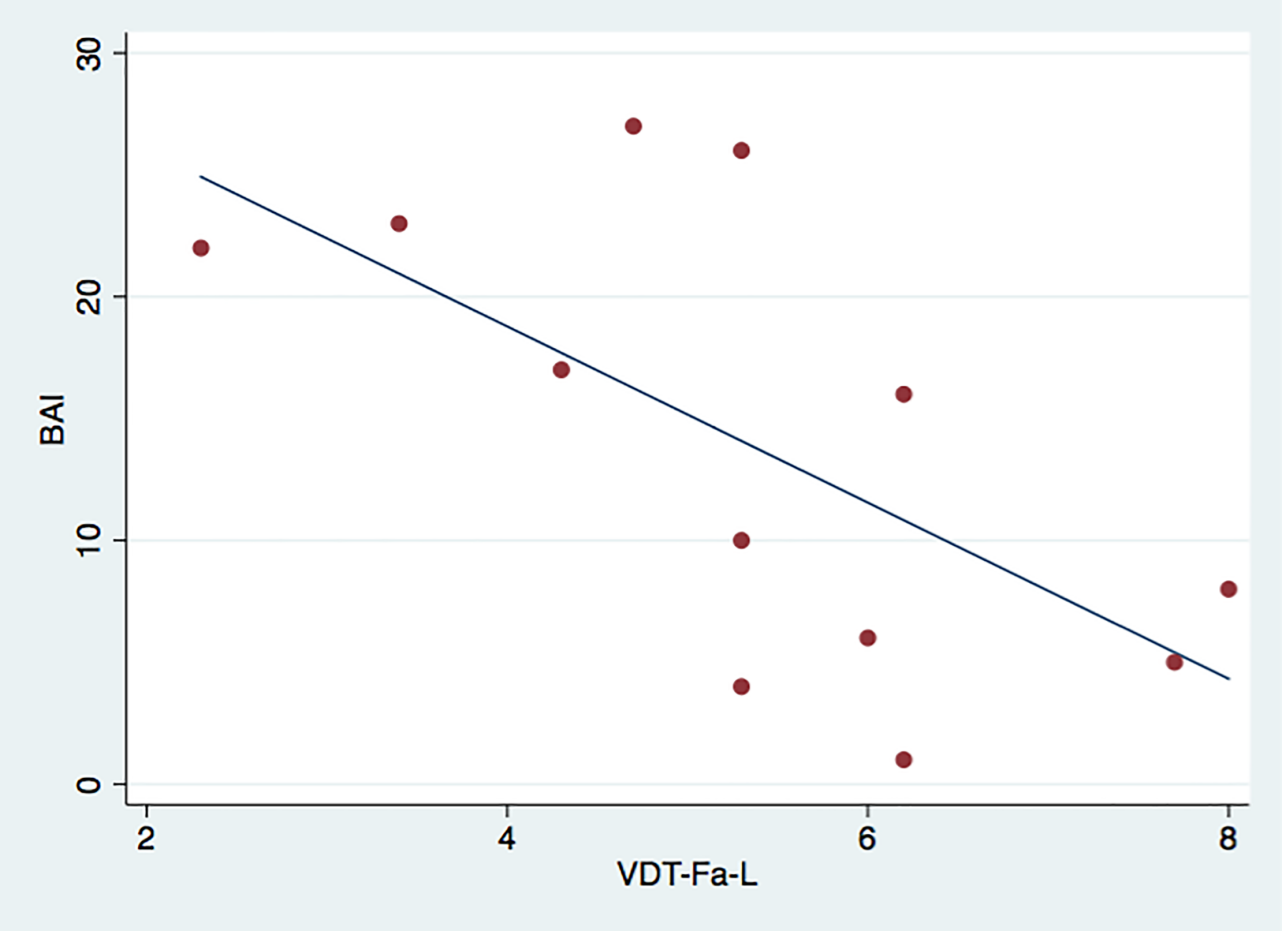
Scatter plot displaying inverse correlation between BAI scores and VDT of left side of face. For visualization purposes, the line illustrates the trend of the relationship between the two variables.

**Fig 5:**
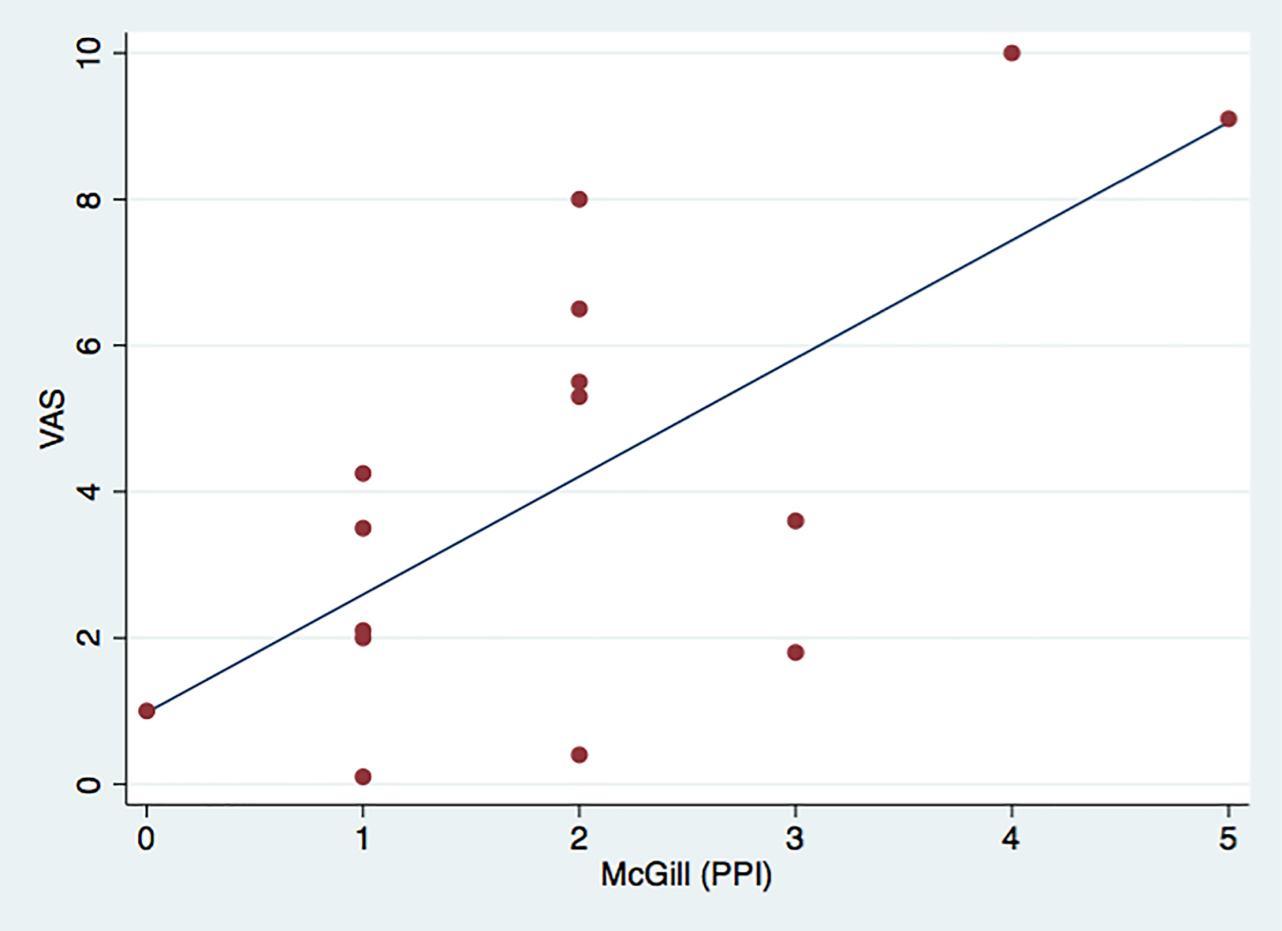
Scatter plot displaying inverse correlation between McGill PPI and VAS. For visualization purposes, the line illustrates the trend of the relationship between the two variables.

Analysis of correlations between the clinical measures of pain and the QST parameters revealed a significant direct correlation only between the scores of the DN4 and the McGill PRI (T) (rs = 0.5354, p = 0.0397, n = 15) and between the VAS and the McGill PPI measures (rs = –0.5824, P = 0.0227, n = 15). On the other hand, a significant inverse correlation was found between the BAI scores and the VDT values for the left side of the face (rs = –0.642, p = 0.024, n = 12). The scatter plots that illustrate these correlations are shown in Figs 3–5.

## Discussion

This study was conducted in Brazilian subjects with a diagnosis of BMS, based on the diagnostic criteria of the ICHD-3. These critera define BMS as “an intraoral burning or dysaesthetic sensation, recurring daily for more than two hours per day over more than three months, without clinically evident causative lesions.” Using this classification, published reports indicate that BMS has a higher prevalence in women within the age range of 60–69 years [6]. Other criteria divide subjects with burning-mouth complaints into two subgroups: BMS (of neuropathic origin) and nociceptive burning mouth. A recent study using the latter classification reported a mean age of 62.30 years for subjects with BMS and 61.58 years for patients with nociceptive burning mouth [31]. The data obtained in the current study (mean age 64.4 years, BMS group comprised of 80% women) agree with the findings of previous studies. In the present study, a meticulous clinical investigation was conducted to avoid the inclusion of subjects with any condition (e.g. chronic pain or systemic disorders) that could affect the results.

The first objective of this study was to evaluate possible changes in two QST parameters (PPT and VDT) in patients with BMS. The QST is an important research tool that has been applied successfully to different pain syndromes [32], and recently in patients with BMS [14, 15, 22]. However, these studies evaluated the presence of changes in the thermal and tactile sensitivity of subjects affected by this condition.

In order to investigate the neural mechanism and the level of neural system dysfunction in BMS, a pioneer study combined the use of QST (through measurement of thermal thresholds) with blink reflex evaluation [14]. The main finding of that study was the heterogeneity of the type of nerve fibers altered in BMS. While some BMS patients exhibited signs of large-fiber neuropathy, others showed signs of small-fiber neuropathy [14]. Another study that investigated whether BMS represents a generalized neuropathy reported hypofunction of Aδ fibers in individuals diagnosed with BMS, specifically within the peripheral distribution of the lingual nerve, a branch of the mandibular nerve [16]. Interestingly, the same study reported an additional hypofunction of C-fibers, detected through VDT performed in the distal extremity of the body [16]. Both Aδ and C fibers mediate deep pain sensitivity, which can be assessed through PPT. So far, no study has reported changes in the PPT of patients with BMS. In the current study, the PPTs on the tongue, right and left sides of the face were higher in patients with BMS compared to healthy volunteers. These results represent changes in the deep sensitivity, indicating a small-fiber (Aδ and C) dysfunction related to BMS. Small-fiber dysfunction has also been described in other neuropathic disorders, such as diabetic neuropathy [33]. This aspect suggests the presence of hypoesthesia, which was evidenced in our study by the increased PPT Z-scores found in patients with BMS (S3 Fig). However, differences in the nature of the tests could have affected the results, considering that previous studies evaluated thermal and tactile sensitivity [14–16] through QST, or the density of the intra-epithelial nerve fibers of the tongue and mucosa, through biopsy [16], while this is the first study that evaluated the PPT in BMS patients.

The current study also showed a significantly lower VDT of the tongue and right side of the face in BMS patients. These results correlate with the findings of a recent study that reported a reduction in the vibration thresholds of the distal part of the feet in patients with BMS [13, 16]. When analyzed collectively, these findings indicate a hypofunction of large fibers (Aß), as is also present in other neuropathies [34, 35].

Variations in the values of the QST parameters may occur depending on the area of the body studied [24, 25]. Therefore, changes in PPT and VDT were investigated in five different (intraoral and extra-oral) regions within the V3 distribution. Our results showed that the specific site of examination also had a major effect on the results. Considering the changes in the blink reflex previously demonstrated in patients with BMS [14], a more-complete investigation of all QST parameters and including other regions of the head as well as the function of other cranial nerves will be necessary to provide more-detailed information regarding BMS pathophysiology.

Considering the classification of BMS as a neuropathic condition, recent studies have also explored the applicability of some questionnaires that are often used in other chronic-pain disorders, such as the MPQ, and specific questionnaires for neuropathic pain, such as the DN4, in this condition. One of these studies validated the use of the DN4 questionnaire as a screening tool for BMS patients, confirming the neuropathic nature of this condition [18]. Nonetheless, another study concluded that the neuropathic-pain questionnaires currently available, such as the DN4, have low sensitivity and specificity in the BMS diagnosis. Therefore, these questionnaires can be used as supplementary tools, always combined with a comprehensive clinical examination of the tongue and oral mucosa in addition to complementary exams [31]. The present study found a direct correlation between the DN4 values and the McGill PRI (T), which confirms the neuropathic character of BMS. A direct correlation between the clinical data, recorded by the VAS, and by the variable PPI of the MPQ questionnaire was found. These latter results were expected, considering that in this study, both the VAS and PPI represented the perception of a burning sensation in the individuals affected. While the VAS represents a subjective assessment of the burning sensation, the PPI provides an objective assessment of the feature.

The present study investigated the levels of depression and anxiety in BMS patients, and their correlation with the PPT and VDT. The presence of higher levels of anxiety and depression has been explored previously in patients diagnosed with BMS. Nevertheless, there is still no consensus on this matter; for instance, the results of one study suggested that patients with BMS suffer more from anxiety than from depression [10], while another epidemiological study, conducted on post-menopausal women, reported that 53.48% of patients with burning mouth have moderate to severe depression, while 46.51% have a mild level of anxiety [36]. In the present study, 58.33% of BMS subjects showed minimal levels of depression, 16.67% displayed mild depression, and 25% moderate or severe depression. At the same time, 66.67% of BMS subjects had minimal or mild anxiety levels. In addition, the depression levels were significantly higher in BMS patients compared to the control group, confirming the findings of a previous study that reported greater severity of depression symptoms associated with BMS [27].

An inverse correlation was found between the BAI scores and the VDT on the left side of the face. Although not significant, a trend toward lower VDT on the left side of the face was demonstrated in patients with BMS. These data raise the hypothesis psychogenic factors could be related to this condition. However, these are the results of a pioneering study, involving the use of QST (PPT and VDT), combined with general pain questionnaires (MPQ) and specific questionnaires used for neuropathic pain screening (DN4), anxiety evaluation (BAI) and depression assessment (BDI) in Brazilian individuals with BMS. Therefore, further research will be necessary to confirm and expand our results. The limited sample size is another factor that should be considered when interpreting the current findings.

## Conclusions

BMS is a neuropathic condition that is difficult to diagnose and to manage clinically, with unclear pathophysiology. The results of this study concord with and expand the findings of recent studies that demonstrated the presence of somatosensory changes present in this disorder, thus enhancing the prospects of future therapeutic strategies. In addition, our results suggest that that psychogenic factors, particularly depression and anxiety, could be associated with BMS. However, future studies are needed to confirm this hypothesis.

## Supporting information

S1 Fig Portuguese version of the McGill Pain Questionnaire. (PDF)

S2 Fig Portuguese version of the DN4 Questionnaire in Portuguese. (PDF)

S3 Fig Z-scores. (PDF)
