# Supplementary material for: Changes in the vibration sensitivity and pressure pain thresholds in patients with burning mouth syndrome
Source: PLoS One. 2018 May 21;13(5):e0197834. doi: 10.1371/journal.pone.0197834 (PMC5962090; doi:10.1371/journal.pone.0197834)

Questionário de Dor McGill

Voluntário: ____________________

IAvD: S_____ Af_____ Av _____ M(S) _____ M(AfAV) _____ M(T) _____ PRI (T) _____ IDA _____

(1-10) (11-15) (16) (17-19) (20) (17-20) (1-2)

1 Espasmódica _
Tremor _
Pulsátil _
Latejante _
Martelante _

2 Crescente _
Repentina _
Provocada _

3 Picada _
Agulhada _
Perfurante _
Punhalada _
Lancinante _

4 Aguda _
Cortante _
Dilacerante _

5 Beliscante _
Pressionante _
Pinçante _
Cãibra _
Esmagamento _

6 Fisgada _
Puxão _
Distensão _

7 Quente _
Queimação _
Escaldante _
Queimadura _

8 Formigamento _
Coceira _
Ardência _
Ferroada _

9 Insensibilidade _
Sensibilidade _

Que Machuca _
Dolorida _
Forte _

10 Suave _
Tensão _
Esfolante _
Rompimento _

11 Cansativa _
Exaustiva _

12 Enjoativa _
Sufocante _

13 Amedrontadora _
Apavorante _
Aterrorizante _

14 Castigante _
Debilitante _
Cruel _
Perversa _
Mortal _

15 Desgraçada _
Enlouquecedora _

18 Incômoda _
Perturbadora _
Desconforto _
Intensa _
Insuportável _

17 Difusa _
Irradiante _

Penetrante _
Que transpassa _

18 Aperto _
Dormente _
Estirante _

Esmagadora _
Demolidora _

19 Fresca _
Fria _
Congelante _

20 Importunante _
Nauseante _
Angustiante _
Desagradável _
Triturante _

IDA

0 Sem dor _
1 Leve _
2 Desconfortante _
3 Angustiante _
4 Horrível _
5 Excruciante _

Breve _
Momentânea _
Transitória _

Rítmica _
Periódica _
Intermitente _

Contínua _
Estável _
Constante _

Interna _
Externa _

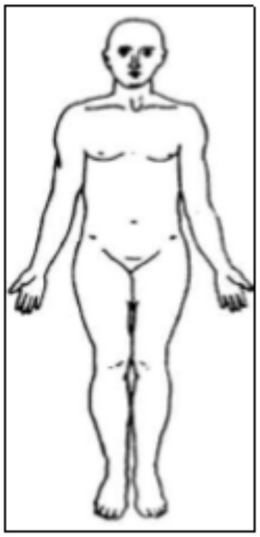

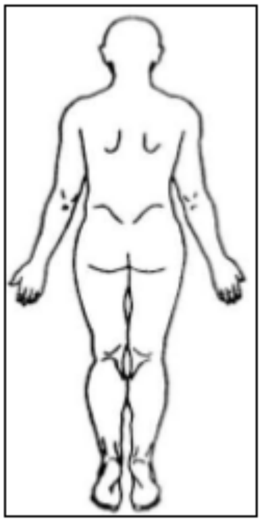

Supplement: S1 Fig — (PDF) [file pone.0197834.s001.pdf]
