# Supplementary material for: Changes in the vibration sensitivity and pressure pain thresholds in patients with burning mouth syndrome
Source: PLoS One. 2018 May 21;13(5):e0197834. doi: 10.1371/journal.pone.0197834 (PMC5962090; doi:10.1371/journal.pone.0197834)
Supplement: S2 Fig — (PDF) [file pone.0197834.s002.pdf]

(Douleur Neuropathique 4)

Voluntário: ___________________________________

Por favor, nas quatro perguntas abaixo, complete o questionário marcando uma resposta para cada número:

ENTREVISTA DO PACIENTE

Questão 1: A sua dor tem uma ou mais das seguintes características?

	Sim	Não
1- Queimação		
2- Sensação de frio dolorosa		
3- Choque elétrico		

Questão 2: Há presença de um ou mais dos seguintes sintomas na mesma área da sua dor?

	Sim	Não
4- Formigamento		
5- Alfinetada e agulhada		
6- Adormecimento		
7- Coceira		

EXAME DO PACIENTE

Questão 3: A dor está localizada numa área onde o exame físico pode revelar uma ou mais das seguintes características?

	Sim	Não
8- Hipoestesia ao toque		
9- Hipoestesia a picada de agulha		

Questão 4: Na área dolorosa a dor pode ser causada ou aumentada por:

	Sim	Não
10- Escovação		

ESCORE

0 – Para cada item negativo 1 – Para cada item positivo

Dor Neuropática: Escore total a partir de 4/10.

( ) Dor Nociceptiva ( ) Dor Neuropática
