# Supplementary material for: Changes in the vibration sensitivity and pressure pain thresholds in patients with burning mouth syndrome
Source: PLoS One. 2018 May 21;13(5):e0197834. doi: 10.1371/journal.pone.0197834 (PMC5962090; doi:10.1371/journal.pone.0197834)
Supplement: S3 Fig — (PDF) [file pone.0197834.s003.pdf]

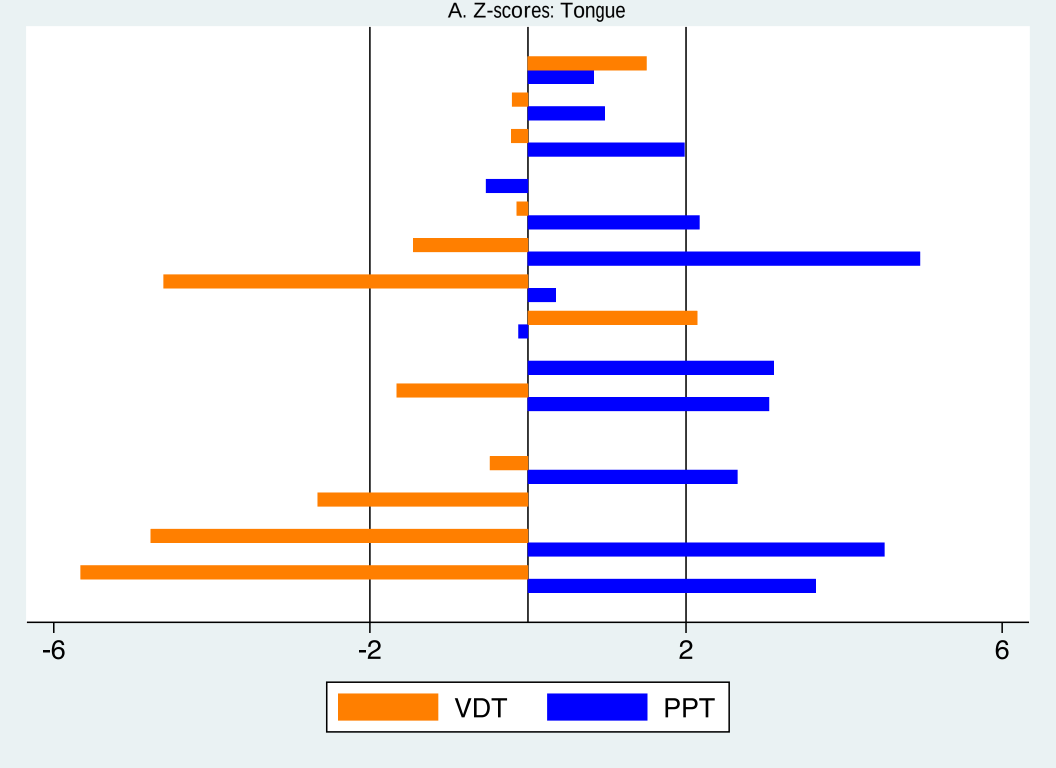
A. Z-scores: Tongue
-6
-2
2
6
VDT
PPT

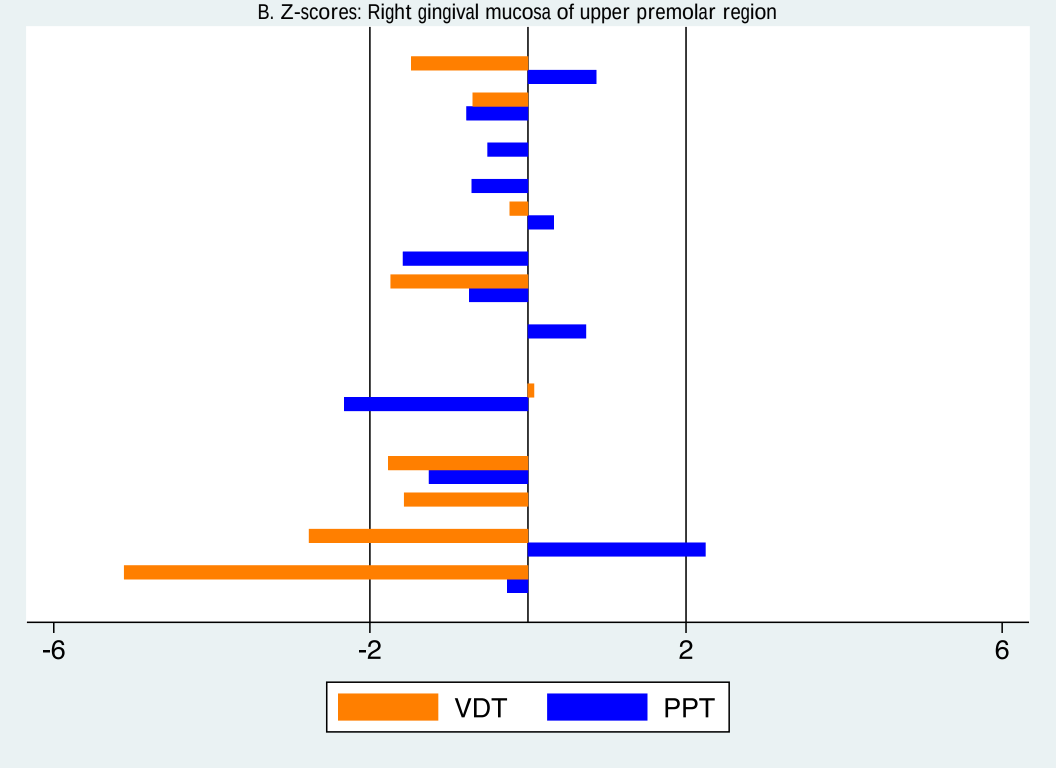

B. Z-scores: Right gingival mucosa of upper premolar region
-6
-2
2
6
VDT
PPT

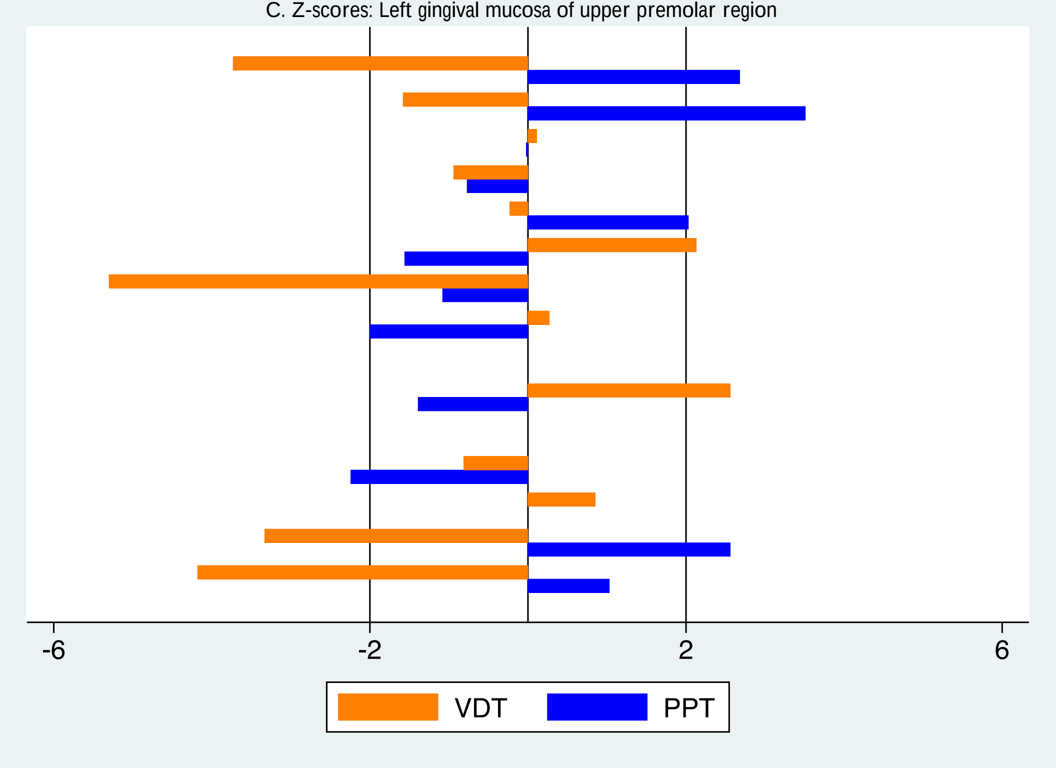
C. Z-scores: Left gingival mucosa of upper premolar region
-6
-2
2
6
VDT
PPT

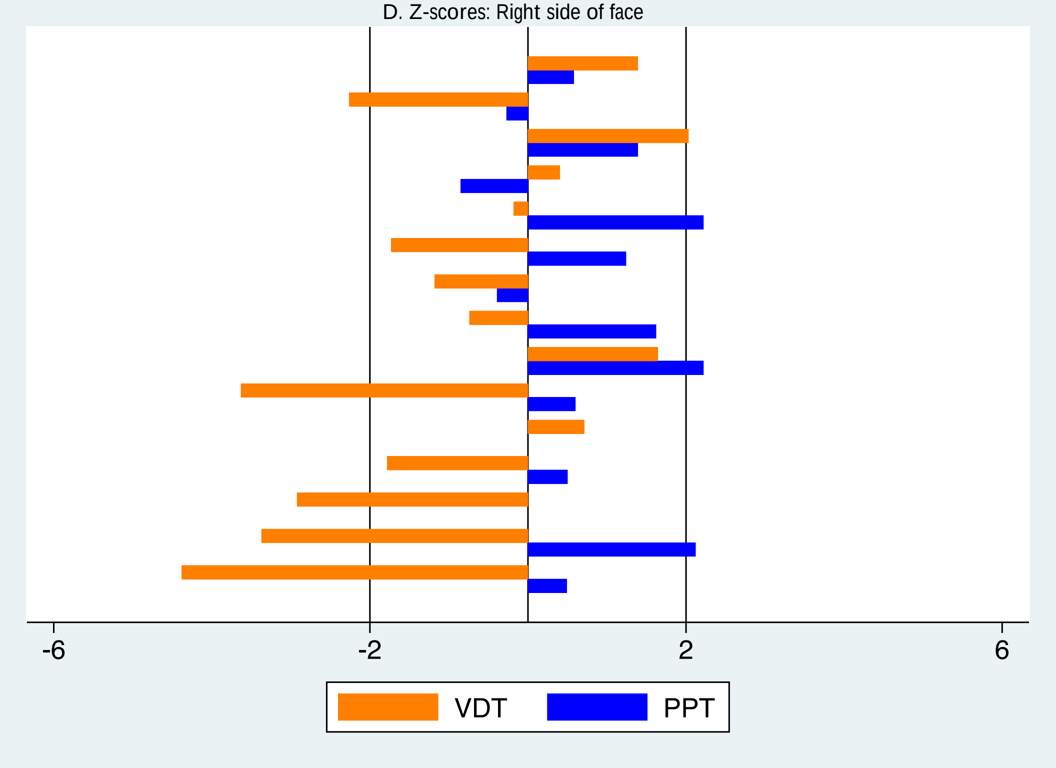
D. Z-scores: Right side of face
-6
-2
2
6
VDT
PPT

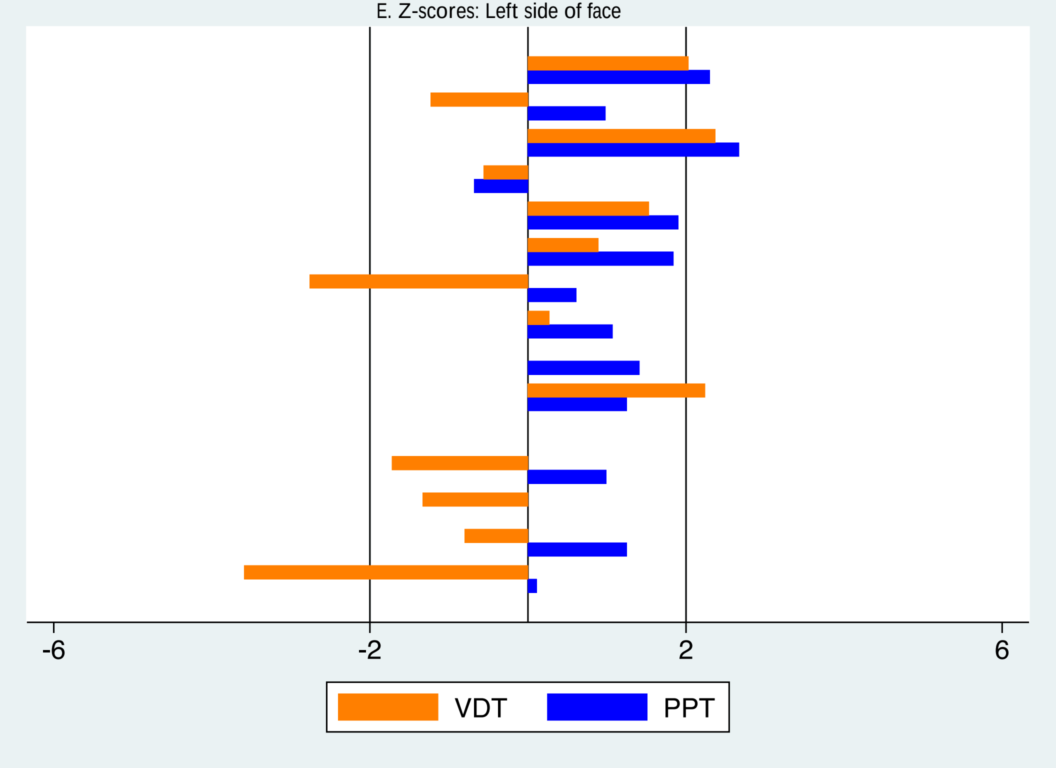
E. Z-scores: Left side of face
-6
-2
2
6
VDT
PPT
